# The effects of COVID-19 on the rehabilitation of persons with aphasia: A scoping review

**DOI:** 10.4102/sajcd.v69i2.920

**Published:** 2022-08-04

**Authors:** Khetsiwe P. Masuku, Gift Khumalo, Nontokozo Shabangu

**Affiliations:** 1 Department of Speech Pathology, Faculty of Humanities, University of the Witwatersrand, Johannesburg, South Africa

**Keywords:** aphasia, COVID-19 pandemic, rehabilitation, telehealth, social participation

## Abstract

**Background:**

The impact of the Coronavirus disease 2019 (COVID-19) pandemic was more pronounced on the well-being of persons with disabilities, especially in low- and middle-income countries. There is documented evidence of the rippling effects of COVID-19 on persons with disabilities. However, not much is known about the impact of COVID-19 on the rehabilitation of persons with aphasia.

**Objective:**

The scoping review explores how COVID-19 affected the rehabilitation of persons living with aphasia.

**Method:**

A scoping review was conducted using Arksey and O’Malley’s framework. A search was conducted on Science Direct, PubMed, Medline, Scopus, ProQuest and Google Scholar, to identify relevant studies published between 2019 and 2022. Data were analysed using thematic analysis.

**Results:**

Most studies regarding the effects of COVID-19 on persons living with aphasia were conducted in the United Kingdom. Five themes emerged from the data, namely, (1) negative impact on rehabilitative care, (2) telehealth and its limitations, (3) impact on social participation, (4) compromised caregiver involvement and (5) mental health challenges.

**Conclusions:**

Findings highlight the need for healthcare professionals to pursue innovative ways in which aphasia rehabilitation and conversational support programmes can be made accessible to persons with aphasia, despite the limitations brought about by a pandemic. Telerehabilitation programmes need to be tailored to the needs of persons with aphasia if they are to be successful. This study highlights the importance and need for the prioritisation of mental health services for persons with aphasia and their caregivers during a pandemic.

## Introduction

Globally, stroke is a leading cause of mortality and disability (Akinyemi et al., 2021; Johnson et al., 2019). The prevalence of people living with the effects of stroke has increased because of the growing and ageing population (Johnson et al., 2019). Stroke is a leading public health issue especially in low- and middle-income countries (LMICs) (Mendis, 2010). Stroke causes more death in LMICs than in high-income countries (HICs) because LMICs are more susceptible to most of the causes of stroke, such as diabetes mellitus, hypertension, human immunodeficiency virus or acquired immunodeficiency syndrome (HIV and AIDS), traumatic aphasia that is caused by interpersonal violence and motor vehicle accidents (Duff, Ntsiea, & Mudzi, 2014; Johnson et al., 2019; Ntsiea, 2019; Sambo & Kirigia, 2014). Akinyemi et al. (2021), Johnson et al. (2019) and Ntsiea (2019) further submit that in LMICs, stroke occurs in younger people with calamitous psychological, social and economic consequences on the individual with a stroke, their family and society.

Stroke is the most common cause of aphasia (Khedr et al., 2020). Approximately a third of stroke survivors experience aphasia (Van Oers et al., 2018). Aphasia is an acquired adult language impairment that affects spoken language expression, spoken language comprehension, written expression, reading and reading comprehension and cognitive nonlinguistic skills (Brady et al., 2016). Language impairments have a significant impact on an individual’s quality of life because of their effect on communication, which is not only a basic human need and right but also a human power (Jokel et al., 2014; Williams, 2000). Ultimately, aphasia has an impact on participation.

Communication intervention becomes imperative in this population (persons with aphasia) because when communication is affected, vocational, family as well as social relations are affected, often leading to frustration, isolation, hopelessness and depression (Hilari et al., 2010; Masuku, Mophosho, & Tshabalala, 2018). The surviving stroke population is increasing, and subsequently there is also an increase in the number of persons living with aphasia. The effects of a language impairment are devastating on the quality of life of both the person with aphasia and their families, thus creating a greater demand for preventive, therapeutic and rehabilitation services at different levels of care (Akinyemi et al., 2021; Stinear, Lang, Zeiler, & Byblow, 2020). In addition to the linguistic challenges, persons with aphasia will experience physical, emotional, psychological and social challenges (Doogan et al., 2018). Thus, a multidisciplinary approach to their rehabilitation is required to holistically address their needs. The rehabilitation teams usually consist of speech language therapists, occupational therapists and physiotherapists (Doogan et al., 2018). Persons with aphasia may also require psychological intervention, as they are likely to suffer from clinical depression and emotional distress as a result of communication difficulties (Santo Pietro et al., 2019).

Research has shown that rehabilitation intervention for aphasia is important because it increases psychosocial well-being (Hilari et al., 2010), communication success (Johansson, Carlsson, & Sonnander, 2012) and participation in everyday life (Johansson et al., 2012). Rehabilitation programmes for persons with aphasia also provide an opportunity for social networks and peer support to persons with disabilities and their families. The World Health Organisation (2017, p. 21) defines rehabilitation as ‘a set of interventions designed to optimize functioning and reduce disability in individuals with health conditions in interaction with their environment’. The British Society of Rehabilitation Medicine (2003) further present that for stroke patients, rehabilitation is a process of active change by which a person who lives with a disability acquires the knowledge and skills needed for optimal physical, psychological and social function. The authors of this study therefore conceptualise rehabilitation using the above-mentioned definitions. Rehabilitation is thus all-encompassing and extends beyond that of addressing body structure and function and activity limitations (Ntsiea, 2019) and should address or alleviate factors that hinder full social integration of the patients, including return to work and improving carer support. The World Health Organisation (2017), in their 2030 strategy which was implemented in 2017, declares rehabilitation as an essential component of integrated healthcare and further states that to achieve sustainable development goal 3 (that of universal health coverage), access to rehabilitation services is necessary. In a South African study sample, all patients who had suffered a stroke received physiotherapy, and only patients from 16 of the 21 health centres received speech therapy and occupational therapy in addition to physiotherapy (Ntsiea, 2019).

Although extensive research shows that rehabilitation is an essential part of the care continuum and positively impacts physical, psychological and social functioning of persons with aphasia, studies suggest that rehabilitation needs remain unmet globally (Akinyemi et al., 2021; Masuku et al., 2018; Ntsiea, 2019; Rhoda et al., 2014; Sherry, 2014; World Health Organisation, 2017). Rehabilitation is still not a health priority, especially in LMICs (Morris et al., 2021). The unmet rehabilitation needs are further compounded in LMICs (Ntsiea, 2019; Rhoda et al., 2014; World Health Organisation, 2017). Global research on aphasia and rehabilitation shows that unmet rehabilitation needs of persons with aphasia may be attributed to various factors. The factors include the shortage of rehabilitation healthcare professionals such as physiotherapists, occupational therapists and speech language therapists (Bernhardt, Urimubenshi, Gandhi, & Eng, 2020; Khoza-Shangase & Mophosho, 2018). Context-specific factors include the cost of travelling to healthcare facilities to access services (Masuku et al., 2018), cultural interpretations of aphasia which influence health-seeking behaviours (Masuku et al., 2018; Penn & Armstrong, 2017), spiritual beliefs (Masuku & Khoza-Shangase, 2018), limited knowledge of rehabilitation services (Aenishanslin, Amara, & Magnusson, 2020; Masuku et al., 2018), limited number of stroke units which are almost exclusively situated in urban areas (Taylor & Ntusi, 2019) and the cost of rehabilitation and transportation to reach rehabilitation services (Aenishanslin et al., 2020; Masuku et al., 2018). Persons with aphasia may have difficulty accessing health care, support groups and intervention programmes that meet their needs (Worrall et al., 2016).

In March 2020, LMICs as well as HICs declared a national state of disaster, as the COVID-19 pandemic continued to spread globally. Since the beginning of the lockdown in March 2020, life as we know it changed drastically. The pandemic and the national lockdown affected the movements of people across different sectors, including healthcare and social welfare (Spinelli & Pellino, 2020). For example, in healthcare, outpatient clinics were suspended, the wearing of masks became mandatory, caregivers accompanying patients for hospital visits were no longer allowed in the hospital and patients were asked not to come in if they presented with COVID-19 symptoms (Spinelli & Pellino, 2020). The COVID-19 pandemic has also caused multidisciplinary team meetings to be suspended or reduced and has further caused a shortage in healthcare resources, which compromises the potential high standard of care given to patients (Spinelli & Pellino, 2020). Telehealth approaches were implemented to facilitate access to aphasia rehabilitation (Jacobs et al., 2021). Given the healthcare, social, psychological and other needs of persons with aphasia, the above-mentioned changes and restrictions that were brought about by the COVID-19 pandemic were therefore bound to compound the already-existing barriers to rehabilitation healthcare services experienced by persons with aphasia and their families. Although persons with aphasia are typically the elderly, aphasia also affects adults, making them vulnerable to the COVID-19 pandemic. The COVID-19 pandemic can prolong the recovery process of persons with aphasia because of the pandemic’s negative influence on aphasia rehabilitation, such as making it difficult for persons with aphasia to start or continue with therapy. It was therefore important to establish how COVID-19 impacted the rehabilitation of persons with aphasia globally. It has been shown that persons with disabilities have been significantly affected and threatened by the evolving health, social and political dimensions of the pandemic (Goggin & Ellis, 2020).

### Aim

The aim of this study is to provide a scoping review of current literature regarding the effect of the COVID-19 pandemic on aphasia rehabilitation.

## Methods

A scoping review was selected because the intention was to synthesise and map out emerging evidence on the rehabilitation of persons with aphasia in the context of compounding circumstances such as COVID-19, which is relatively new (Arksy & O’Malley, 2005; Levac, Colquhoun, & O’Brien, 2010; Pham et al., 2014). The intention of a scoping review in this area is to provide an overview of existing literature, to identify existing gaps for further research and for the findings of the review to influence public policy and guide clinical practice. Findings may also contribute to the strengthening of rehabilitation services in the context of a pandemic and inform the improvement of new adapted methods of rehabilitation that may have been implemented because of COVID-19.

### Design and steps followed in the review process

An interpretive scoping literature review employing Arksey and O’Malley’s (2005) scoping review methodology was used. In accordance with this framework, the following steps were followed:

1. The initial research question was developed by K.M., G.K. and N.S.
2. Relevant studies that answer the research question were identified using various search strategies and databases to avoid bias (Schlosser et al., 2005).
3. Relevant studies that met the inclusion and exclusion criteria were included in the analysis.
4. Data were charted using a narrative descriptive analytical framework.
5. Data were then collated and summarised.

Levac et al. (2010) state that a scoping review is broad in nature, as the focus is more on the breadth than it is on the depth of literature, and thus it becomes necessary to clearly define the scope of the inquiry through a clearly defined population (aphasia patients), a clearly stated concept or interest (rehabilitation) and specified context (COVID-19) to ensure a successful search strategy. In addition to the above, a clear inclusion and exclusion criteria was set and agreed upon by all three reviewers to prevent selection bias (Gough et al., 2012). Table 1 presents the inclusion and exclusion criteria for articles.

**TABLE 1:** Criteria for selecting studies.

Criteria terms	Inclusion	Exclusion
Defining the concept: rehabilitation	Include publications on the rehabilitation of persons with aphasia	Exclude publications focusing on the provision of neurological services, not rehabilitation
Target population: persons with aphasia	Include publications of adults who have had aphasia as a result of stroke	Exclude publications focusing on other neurological conditions resulting from stroke
Context: during COVID-19	Include publications on the rehabilitation of persons with aphasia during the COVID-19 pandemic, published between 2019 and 2022	Exclude publications on the rehabilitation of persons with aphasia but not focused on COVID-19
Other	Include only full-text articles published in English	Exclude articles written in languages other than English and also not available in English

### Data sources and search strategy

A systematic search was conducted in January 2022 by three reviewers who are all rehabilitation healthcare professionals. Two of the reviewers are speech language therapists who manage and conduct research on and with persons with aphasia and one reviewer is a social worker who has an interest in disability research. Levac et al. (2010) advise that ideally, experts in the research subject and the scoping research methodology should be involved in scoping reviews to increase rigour.

**FIGURE 1:**
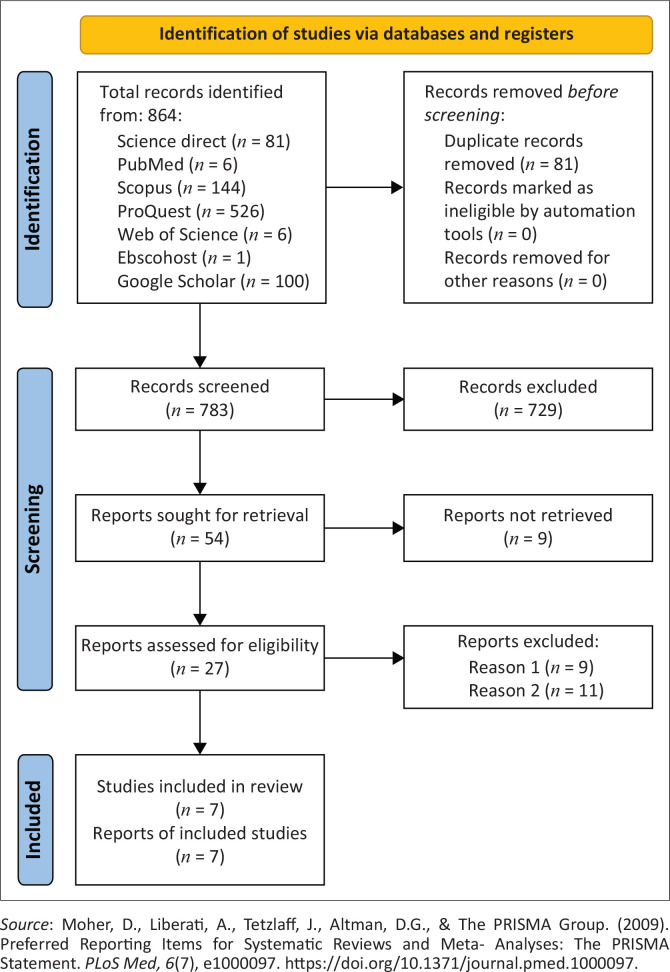
Preferred reporting items for systematic reviews and meta-analysis flow diagram for included publications.

Three search strategies were used to ensure that all available literature on the subject were included (Davis, Drey, & Gould, 2009), namely (1) online database searches, (2) general Internet browser via Google Scholar and (3) hand searches. Multiple electronic databases were utilised during the search (Bramer 2016). A total of seven electronic databases accessible through the University of the Witwatersrand library were included in the search, namely Science Direct, PubMed, Scopus, ProQuest, Web of Science, Ebscohost and Google Scholar. Google Scholar often reports a very high number of hits compared to other databases (Bramer, 2016). Therefore, based on the feasibility and time required to screen each hit and because the authors believed that screening further would not produce articles relevant to the study, a theoretically informed decision was made to screen only the first 100 hits as sorted by relevance, as proposed by Haddaway, Collins, Coughlin and Kirk (2015). An additional hand search, where the two reviewers screened through the reference lists of the identified studies, was conducted to make sure that no relevant studies were missed (Gough et al., 2012). The electronic databases and university library websites contained similar search terms and publication requirements. The Boolean search conducted in each individual database and university website used the following search terms: ‘aphasia patients’ AND ‘rehabilitation’ AND ‘covid’. Figure 1, an adapted Preferred Reporting Items for Systematic Reviews and Meta-Analyses (PRISMA) (Moher et al., 2010), illustrates the search and article selection process followed during the study.

### Citation management and article screening

All publications were imported into Mendeley, a web-based reference management programme. The Mendeley web-based reference management programme was used to facilitate a rigorous process of data management (King et al., 2011). The authors K.M. and G.K. were responsible for searching and screening the articles. Once the articles were imported onto Mendeley, duplicates were removed manually by K.M. and G.K. From there, the screening of the remaining publications was conducted collaboratively by K.M. and G.K., a process that ensured that the screening process was conducted by at least two authors (Blanco et al., 2019). Articles that did not met the minimum inclusion and exclusion criteria were removed first at title and then at abstract level. At both the title and abstract level, the inter-rater agreement was 100%. Where discrepancies of the saved publications from the collaborative screening arose, they were discussed by the two authors until consensus was reached. Full-text articles that were deemed relevant for the study post the title and abstract level were then again reviewed collaboratively by K.M. and G.K. Inter-rater agreement at the article screening level was 80%, a percentage that is deemed acceptable by Graham, Milanowski and Miller (2012). Disagreements were discussed by K.M., G.K. and an independent reviewer who is well versed on review methodologies until consensus was reached. Data from the full-text articles were characterised into a spreadsheet and charted according to the following headings: author and year of publications, title, focus, aims, methodology and results. Once data were charted, inductive thematic analysis was employed. The analysis followed the steps proposed by Braun and Clarke (2006). An overview of the characteristics of the seven articles that are included in the review is provided in Table 2.

**TABLE 2:** Studies with implications for rehabilitation of persons with aphasia.

Author(s) (date)	Publication title	Publication focus and aims	Methodology	Context	Results
Zaidi et al. (2020)	Malaysia Stroke Council guide on acute stroke care service during COVID-19 pandemic	Not stated	Position paper	Malaysia	Patients are unable to access poststroke rehabilitation facilities and programmes thus hindering the rehabilitation progress.
Migliocchio et al. (2021)	Management of an aphasic-apraxic patient during the COVID-19 pandemic: a case report.	The aim of the study was to describe the rehabilitation method applied to a patient with predominantly anarthric impairment and the results obtained from the application of this methodology both in the acute as well as in the sub-acute phase	Case study research	Italy	As a result of the emergency, COVID-19 training during the acute phase was conducted every other day by only two different specialists. Caregivers were included in the patients’ treatment via computer media remotely. Video and audio-visual media were implemented to demonstrate oral related exercises because the visual feedback needed for this type of commitment could not be provided because of the PPE, made necessary by the ongoing pandemic.
Kong (2021)	The impact of COVID-19 on speakers with aphasia: what is currently known and missing?	The study aimed to provide an understanding of the impact of COVID-19 on aphasia, telerehabilitation for aphasia, support initiatives, available resources and recommendations for clinical practice	Viewpoint paper	United States of America	Reduced social participation and sense of social connectedness. Difficulty participating in online therapies because of hemiparesis or other motor difficulties; co-existing age and cognition-related problems. Bilingual and multilingual populations may experience difficulties with interactions with clinicians in online platforms. Persons with aphasia who were enrolled in intervention programmes with set timelines or schedules have been affected because of the abrupt suspension or termination of speech and language therapy. Rehabilitation programmes involving multi-intervention or multidisciplinary approaches, which are fairly common for persons with aphasia to participate in community outpatient clinical settings. Negative effects of quarantine on healthcare professionals. Clinicians’ lack of knowledge and experience with conducting therapy in online platforms.
Pandian et al. (2021)	Maintaining stroke care during the COVID-19 pandemic in lower- and middle- income countries: World Stroke Organisation Position Statement endorsed by American Stroke Association and American Heart Association	Produce pragmatic recommendations on methods to reserve the existing SSOC during COVID-19 in LMIC. Adapt existing stroke care workflows in a manner that both protects healthcare workers whilst still offering timely acute reperfusion therapy to as many patients as is feasible. Propose best stroke practices that may be low-cost but high-impact and commonly shared across the world.	Position statement, informed by a literature search on PubMed and Google Scholar	United Kingdom	Poststroke rehabilitation has been affected because of the restrictions in travel and because of the physical distancing that has to be maintained during therapy. A need to reduce the duration of physical contact during each session of neurorehabilitation. Telerehabilitation is a feasible alternative to physical rehabilitation in this COVID-19 era.
Kong (2021)	Mental Health of Persons with Aphasia during the COVID-19 Pandemic: Challenges and Opportunities for Addressing Emotional Distress.	To summarise the challenges posed by COVID-19 to the management of aphasia.	Position paper	United States of America	Persons with aphasia have experienced various levels of emotional distress about COVID-19 and its impact on them, their loved ones and their communities. Some persons with aphasia might not be able to transition to teletherapy amidst COVID-19 because of digital inequality. Limited aphasia apps which are still available predominantly in English. Caregivers of persons with aphasia assumed multiple important roles in the rehabilitative process and were overwhelmed.
Lucas et al. (2021)	Impact of COVID-19 on the stroke rehabilitation pathway: multidisciplinary team reflections on a patient and carer journey from acute to community stroke services	To report on the multidisciplinary teams’ ways of working to accommodate the patient’s priorities whilst negotiating the COVID-19 pandemic	Case presentation	United Kingdom	The COVID-19 pandemic has posed significant challenges to the quality of the patient’s rehabilitation recovery and quality of life. The pandemic and the national lockdown in the UK have contributed towards increased isolation for the patient and his partner, reduced availability of external support services and inability to create peer support networks. The case also highlighted the potential benefits of shared goal setting for patients with communication difficulties and transdisciplinary (i.e. integrating speech therapy and psychology) approaches to community stroke rehabilitation.
Chadd, Moyse and Enderby (2021)	Impact of COVID-19 on the speech and language therapy profession and their patients	To review the changes to speech language therapy services triggered by the COVID-19 pandemic with respect to referral rates, service delivery and outcomes, as well as examining the contribution of SLTs to the neurorehabilitation of COVID-19 patients.	Mixed methods research	United Kingdom	Referrals to SLT services during the acute COVID-19 period in the United Kingdom were substantially less than in the same period in 2019. A number of service changes were common, including adopting more flexible approaches to provision (such as teletherapy) and being unable to provide services to some patients. Database analysis suggests fewer patients accessed SLT since the pandemic began, including a reduction in neurorehabilitation patients. For those who received SLT, the outcomes did not change. SLTs supported a range of needs of COVID-19 patients. Treatment outcomes for COVID-19 patients with dysphagia were positive.

SSOC, stroke systems of care; LMIC, low- and middle-income countries; PPE, personal protective equipment; SLT, speech language therapy.

### Ethical considerations

Ethical clearance to conduct this study was obtained from the Human Research Ethics Committee (Non- Medical), reference number: STA_2021_41. The articles accessed and reviewed are in the public domain and thus the authors did not need permission to conduct the review. This study did, however, follow all ethical standards for a research without direct contact with human or animal subjects.

## Results

### Descriptive information on the studies that were included

Methodologies used in the seven studies included one qualitative (14%), one mixed methods study (14%), one case presentation (14%) and four opinion and position statements (58%). The qualitative design consisted of one single-subject design (*n* = 1), whilst the mixed methods design consisted of two surveys conducted at two different periods. The first survey was with (*n* = 544) speech language therapists whilst the second was with (*n* = 413) speech language therapists. Geographically, three (43%) of the studies were conducted solely or in collaboration with researchers from the United Kingdom, 29% (*n* = 2) were situated in the USA, 14% (*n* = 1) in Italy and 14% (*n* = 1) in Malaysia.

Thematic analysis of the seven studies included in the current study revealed that because of the inability to access full rehabilitation services as a result of the strained healthcare system, lockdown and social distancing restrictions, the rehabilitation and clinical progress of persons with aphasia was compromised (Table 3). The restrictions related to COVID-19 also compromised the social participation of persons with aphasia, some of which is facilitated through aphasia conversational support groups during rehabilitation therapy. Caregiver involvement, which is crucial to aphasia patient care, was also limited because of social distancing restrictions. Telehealth became the preferred alternative of providing face-to-face rehabilitation clinical services. However, even though telehealth facilitated the provision of rehabilitation services during the pandemic, telehealth presented with challenges as a result of patients’ physical limitations, clinicians’ knowledge and skills of using online platforms to conduct therapy and the social isolation of patients. The COVID-19 pandemic affected the mental health of both patients and rehabilitation healthcare professionals, which in turn negatively influenced rehabilitation healthcare services.

**TABLE 3:** Themes and subthemes that emerged from an analysis of the studies included in the review.

Themes	Subthemes	Subthemes
Negative impact on rehabilitative care	Compromised rehabilitation progress	Inability to access services because of lockdown restrictions. Acute rehabilitation conducted every other day because of staff shortage. Number of multidisciplinary team members limited because of social distancing. Abrupt suspension of speech therapy services. Decreased duration of physical contact with patients. Suspension of conversational group therapies.
Telehealth and its limitations	Increased use of telehealth	Video and audio-visual media used to demonstrate oral-related exercises because of mandatory personal protective equipment. Telerehabilitation is a feasible alternative to physical rehabilitation in this COVID-19 era.
	Challenges of telehealth	Difficulty participating in online therapies because of hemiparesis or other motor difficulties, co-existing age and cognition-related problems. Bilingual and multilingual populations may experience difficulties with interactions with clinician in online platforms. Clinicians’ lack of knowledge and experience with conducting therapy in online platforms.
Impact on social participation	Increased isolation	Increased isolation for the patient and their partners.
	Compromised social participation	Reduced availability of external support services. Inability to create peer support networks.
Compromised caregiver involvement	Caregivers were included in the patients’ treatment via computer media remotely. Caregivers of persons with aphasia assumed multiple important roles in the rehabilitative process and were overwhelmed.	-
Mental health challenges	Negative effects of quarantine on healthcare professionals. Caregivers of persons with aphasia assumed multiple important roles in the rehabilitative process and were overwhelmed. Reduced social participation and sense of social connectedness.	-

## Discussion

Findings of the scoping review revealed a scarcity of literature pertaining to the influence of COVID-19 on the rehabilitation of persons with aphasia in HICs as well as LMICs. There was evident dearth of literature on this subject in the African context, despite the high prevalence of stroke and aphasia, exacerbated by constrained access to rehabilitation and strained healthcare resources (Johnson et al., 2019; Ntsiea, 2019; Sherry, 2014). Of the publications that were deemed relevant for the study, a few discussed rehabilitation and aphasia independent of stroke, and the emphasis is on multidisciplinary rehabilitation as opposed to just reporting on speech language pathology. This is because of the close association of stroke and aphasia (Khedr et al., 2020). By its nature, a stroke usually affects different areas including motor, communication, psychological and cognitive abilities, often impacting on participation and quality of life of the stroke survivor and their facilities. Therefore, a team approach to rehabilitation is key.

An analysis of literature revealed that COVID-19 negatively impacted on the rehabilitation and the therapy progress of persons with aphasia, both at the acute and chronic stages, because of regulations imposed by states to curb the spread of the virus. Regulations related to lockdown and social distancing resulted in the inability of persons with aphasia to travel to healthcare facilities to access healthcare. The number of rehabilitation sessions as well as the number of team members making up the multidisciplinary team were significantly reduced to ensure social distancing and to decrease the exposure to the virus. Some healthcare facilities abruptly suspended speech therapy services. Chadd, Moyse and Enderby (2021) agree with the findings of this study that the number of sessions were reduced in that the number of referrals to speech language therapists during the period of 2019 and 2020 in the United Kingdom drastically went down as a result of disruptions to the provision of rehabilitation services by the COVID-19 lockdown.

Although there is evidence of an exponential increase in the use of telerehabilitation as an alternative to physical rehabilitation during COVID-19 (Fu, Burger, Arjadi, & Bockting, 2020), online therapy with persons with aphasia was not always successful. Prior to COVID-19, even though teletherapy was an available option, not all clinicians were using teletherapy. Therefore, when COVID-19 struck, not all rehabilitation healthcare professionals (including speech language therapists) had the necessary knowledge and skills to conduct therapy on an online platform. The success of telerehabilitation services is dependent not only on the client and type of rehabilitation therapy provided, but also on the therapists conducting the intervention (Ebrahim et al., 2021). Therefore, it not surprising that the lack of knowledge and skills of therapists could negatively influence access to rehabilitation for aphasia patients. This finding is also consistent with the findings from a study that was conducted by Eikelboom and Swanepoel (2016) where it was revealed that less than a quarter of healthcare professionals in the study had used teletherapy to conduct therapy with clients.

As previously mentioned, persons with aphasia may present with hemiparesis or other motor difficulties, and they may also present with co-existing age and cognition-related problems. Therefore, to ensure the success of telerehabilitation, therapists must take into consideration the recommendations by Hall, Boisvert and Steele (2012), who propose that before assigning patients to telerehabilitation, therapists need to be aware of factors such as their communication and motor abilities, severity, chronicity and symptoms of aphasia. A study conducted by Neate, Kladouchou, Wilson and Shams (2022) agrees with the finding of this study regarding online therapy as not always being successful. Neate et al. (2022) found that persons with aphasia were often distressed in online sessions for numerous reasons, including experiencing fatigue when they had to speak, challenges around framing of props and videoconferencing platforms putting group members in small tiles making it difficult to read other members’ facial expressions and turn-taking challenges. One of the key outcomes of aphasia therapy is to facilitate communication and increase participation, which is usually facilitated through group-based therapy because it provides natural and social communication (Fama, Baron, Hatfield, & Turkeltaub, 2016). Group therapy also facilitated peer support networks for both the persons with aphasia and their caregivers. Findings in the study, however, reveal that COVID-19 restrictions negatively impacted on support groups, which resulted in patients and caregivers being isolated because they lacked opportunities to engage with others. Lucas et al. (2021) support the findings of this study in that persons with aphasia were unable to access local support groups for peers, thus impacting on their ability to create peer support networks. Additionally, this finding is also supported by a study conducted by Hack (2021) where persons with aphasia expressed feeling lonely, missing their aphasia group therapy, having difficulties with communication and worrying about losing their group members to COVID-19 after the abrupt end to their group therapy sessions.

Shafer, Shafer and Haley (2019, p. 635) submit that ‘caregivers are an integral part of the recovery process for people with aphasia, helping them to navigate outpatient rehabilitative care and reintegrate into the community after hospitalization’. However, findings of this study suggest that during COVID-19, in the acute phase, COVID-19 regulations may have not always allowed caregivers to be physically present during the treatment of persons with aphasia, with some caregivers required to join sessions remotely via telehealth. Where caregivers were involved, they took up multiple roles and as a result they felt overwhelmed, which may have had a negative impact on their mental health. Lucas et al. (2021) agree with this finding in that hospital visitations by caregivers of persons with aphasia were suspended and that the caregivers were therefore unable to be involved in the rehabilitation process of the patients. This affected the functioning of the caregivers as they felt isolated and unable to observe the progress made by their loved ones. In a study conducted in the USA with caregivers of stroke survivors in an inpatient rehabilitation where visitations were suspended (Sutter-Leve, Passint, Ness, & Rindflesch, 2021), it was revealed that caregivers were concerned about the rehabilitation of their loved ones in their absence and expressed uncertainty about their loved ones’ rehabilitation progress and being unable to observe them during therapy and nursing care. Additionally, the restrictions of the COVID-19 pandemic put strain on the caregivers and their loved ones’ relationships (Bakas & Commiskey, 2021). This finding is further agreed upon in a study by Lee et al. (2021) in that the caregivers expressed that they were physically exhausted and were experiencing psychological distress because of concerns about exposing their loved ones to COVID-19, the additional workload and taking up the role of rehabilitation therapists during the pandemic.

## Conclusion

Findings from the study suggest the need for rehabilitation healthcare professionals to pursue innovative ways in which aphasia rehabilitation support and conversational programmes can still be made accessible to persons with aphasia despite the limitations brought about by COVID-19 or any other pandemic or circumstance that may hinder face-to-face patient contact. Group rehabilitation therapy is important for facilitating conversations as well as overcoming conversational barriers that persons with aphasia and their families experience. Conversational groups create a social interactive environment which is ideal for practising communication and participation, which are good for the emotional and mental well-being of persons with aphasia and their families.

Telerehabilitation provides an alternative means to continue patient rehabilitation care, including online aphasia intervention and conversational groups. However, in order for telerehabilitation to be successful, special consideration need to be made for the physical and communicational needs of the person with aphasia and their family. The authors would go as far as to argue for contextual relevant consideration such as data costs, connectivity challenges because of poor infrastructure, health and digital literacy, which are mostly experienced in LMICs. The above-mentioned factors mostly experienced in LMICs may not have come through in the results of this study because of the paucity of literature of the subject as it pertains to aphasia rehabilitation during the pandemic. However, they are a reality in these contexts, and they have the potential to impede the delivery of rehabilitation services. Studies on the subject from LMICs are needed so as to shed some light onto the current telerehabilitation trends when managing persons with aphasia so that context-specific solutions can be forged.

As the global need for telerehabilitation continues to increase, it is therefore necessary to have a rehabilitation healthcare workforce that possess the necessary knowledge, skills and experience, in telerehabilitation. There is a need for renewed and deliberate efforts to integrate telerehabilitation into the academic curriculum for current and future rehabilitation healthcare students. Current rehabilitation healthcare professionals can enhance their telerehabilitation skills through continued professional development activities.
